# Shear wave versus strain elastography in the differentiation of benign and malignant breast lesions

**DOI:** 10.3906/sag-1905-15

**Published:** 2019-10-24

**Authors:** Aykut GÜRÜF, Mesut ÖZTÜRK, İlkay Koray BAYRAK, Ahmet Veysel POLAT

**Affiliations:** 1 Radiology Clinic, Ordu State Hospital, Ordu Turkey; 2 Department of Radiology, Faculty of Medicine, Ondokuz Mayıs University, Samsun Turkey

**Keywords:** Breast, diagnostic performance, shear wave elastography, strain elastography, ultrasonography

## Abstract

**Background/aim:**

To evaluate and compare the diagnostic performances of shear wave elastography (SWE) and strain elastography (SE) in the differentiation of benign and malignant breast lesions.

**Materials and methods:**

The current study included 87 breast lesions in 84 patients. The Breast Imaging Reporting and Data System (BIRADS) categories were determined with ultrasound features. The maximum shear wave velocity (SWV), mean SWV, maximum SWV to fat SWV ratio, and mean SWV to fat SWV ratio were measured using SWE. The strain ratio (SR) was calculated as the ratio of lesion strain to the adjacent fat strain using SE. Receiver operating characteristic (ROC) curves were constructed to assess and compare the diagnostic performances of each parameter.

**Results:**

Forty-five benign and 42 malignant lesions were diagnosed. The sensitivity and specificity of the BIRADS classification was 100% and 35.6%, respectively. Selecting a cutoff SR value of 3.22 led to an 88.1% sensitivity and an 88.4% specificity (AUC: 0.913 [95% CI: 0.854–0.971], P < 0.001). Selecting cutoff maximum SWV value of 3.41 m/s led to an 88.1% sensitivity and an 86.7% specificity (AUC: 0.918 [95% CI: 0.858–0.978], P < 0.001). The diagnostic performance of the maximum SWV, mean SWV, and maximum SWV to fat SWV ratio were similar to the diagnostic performance of the SR (P = 1.00, P = 1.00, P = 0.629, respectively).

**Conclusion:**

SE and SWE are both feasible imaging modalities in the differentiation of malignant and benign breast lesions with similar diagnostic performances.

## 1. Introduction

Ultrasound (US) is one of the most widely used imaging modalities for the early diagnosis and management of breast cancer. While this method was initially used to distinguish cystic masses from solid ones, high frequency transducers, advancements in imaging technology, and the use of the American College of Radiology Breast Imaging Reporting and Data System (BIRADS) in clinical practice has helped in differentiating breast lesions. However, US still has some limitations, such as being an operator-dependent technique with low specificity [1]. Combining elastography with grayscale US findings has been shown to improve the diagnostic accuracy of breast lesions [1,2]. 

Elastography is an US-based imaging modality that evaluates the stiffness of soft tissues by measuring the degree of distortion under pressure [3–6]. Two different techniques have been described, depending on the source of mechanical compression to the examined tissue: shear wave elastography (SWE) and strain elastography (SE). In SE, a mechanical force is applied by the operator to deform the tissue and the tissue strain is assessed. The higher the strain the softer the lesion, and the lower the strain the harder the lesion. As the mechanical compression force applied to the tissue cannot be measured accurately, the absolute tissue strain cannot be calculated. Tissue strain is calculated relative to the adjacent tissues. A strain ratio (SR) is calculated by dividing the strain of a nearby reference tissue to the strain of the examined tissue [7]. A higher SR means stiffer examined tissue. Major limitations of this method include being an operator-dependent technique, having a low reproducibility, high interobserver variability, and providing qualitative or semiquantitative information [8–10].

In contrast to SE, SWE evaluates tissue stiffness through an acoustic radiation force (acoustic radiation force impulse, or ARFI) emitted from the US probe instead of mechanical compression. This acoustic force causes horizontal displacements in the tissue, which are called shear waves. These shear waves contain quantitative data about the elastic properties of the tissue that can be measured in meters per second (m/s) [11]. SWE has the advantages of being more objective, having a higher reproducibility, and having decreased operator dependence [1].

There are limited studies in the current literature investigating whether SWE or SE is more reliable in differentiating malignant and benign breast lesions [12–15]. The present study aimed to assess and compare the diagnostic efficacy of SWE and SE for the differentiation of benign and malignant breast lesions by applying both techniques on the same breast lesions. We also present a brief review of the previously published studies comparing these two elastography techniques.

## 2. Materials and methods

The current study was conducted between June and December 2015 with the approval of our institutional ethics committee. The relevant review board approval code: Ondokuz Mayıs Universitesi Klinik Araştırmalar Etik Kurulu, B.30.2.odm.0.20.08/1800. We obtained informed consent from every participating patient before each examination. The standards for the Reporting of Diagnostic Accuracy Studies guidelines were used [16].

## 2.1. Patients

In total, 87 breast lesions in 84 consecutive women who had been scheduled to undergo US-guided core needle biopsies were studied. Lesions were examined with B-mode US, SWE, and SE before biopsy. The mean age of the study cohort was 49.55 ± 14.57 (range: 21–93) years. The enrollment criteria were as follows: 1) masses that were solid or almost solid (less than 20% cystic component); 2) no history of chemotherapy or radiotherapy for any other malignancies; 3) no history of previous breast cancer; 4) no history of previous biopsy or fine needle aspiration of the lesion.

## 2.2. B-mode US examination

B-mode US, SWE, and SE examinations of the lesions were performed by a radiologist (IKB) with 15 years of experience in breast US, 2 years of experience in breast SE, and 2 years of experience in breast SWE. The examinations were performed on the same day within a time interval of less than 30 min. US and SWE examinations were performed using the Siemens ACUSON S2000 US system (Siemens Medical Solution, Mountain View, CA, USA) with a 9L4 multi-D probe. Patients were placed in the supine position with a raised ipsilateral arm over their head, and they were rolled slightly with the help of a wedge under their shoulder to spread the breast evenly. During the B-mode examination, the maximal lesion size and sonographic features were noted, and the lesions were categorized according to the lexicon of the American College of Radiology BIRADS classification [17]. For patients with multiple masses, every lesion was examined separately and each BIRADS score was determined.

## 2.3. Shear wave elastography

**Figure 1:**
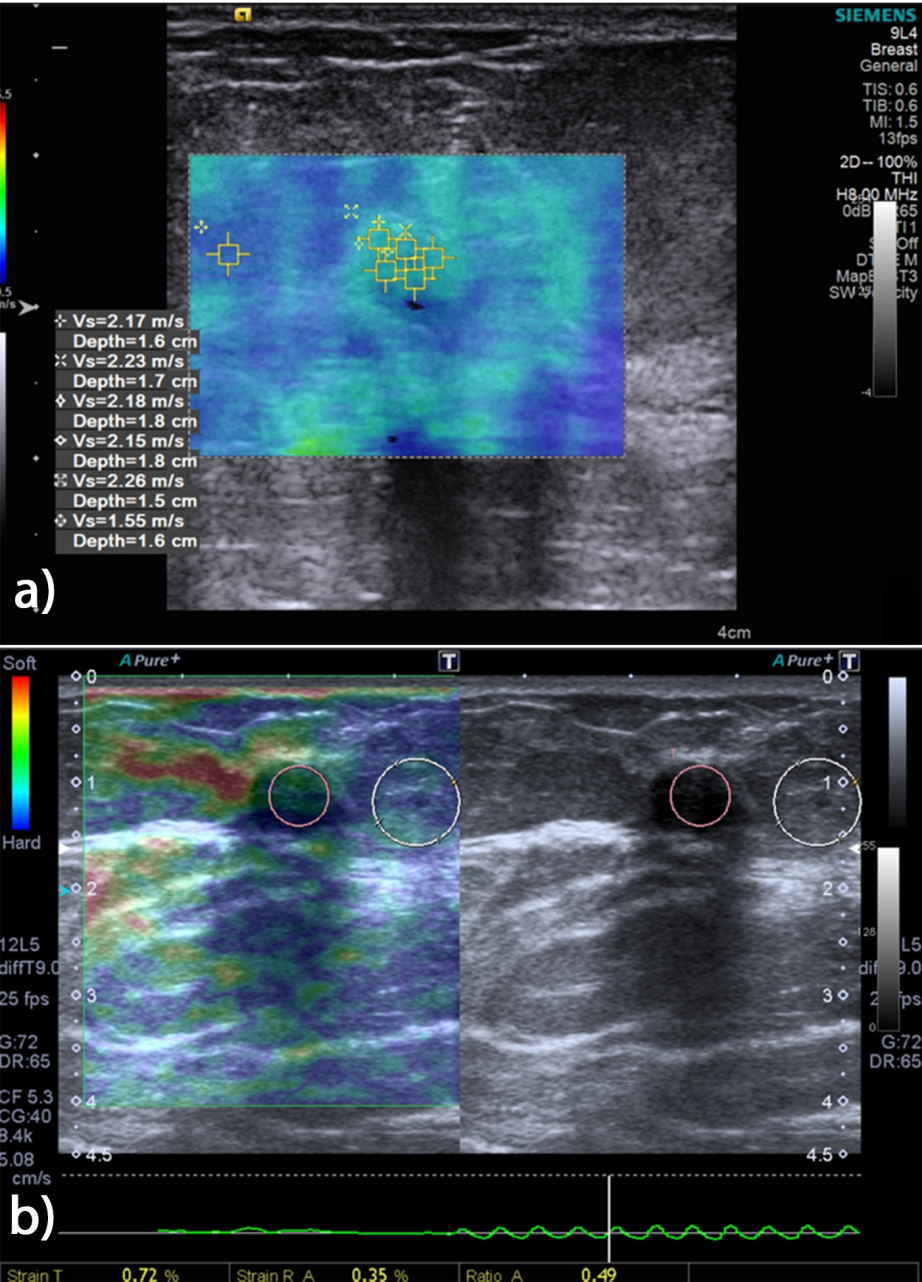
A 54-year-old female patient with a breast mass located in the upper lateral quadrant of the right breast. The lesion was oval, hypoechoic, and measured 11 × 6 mm with its long axis parallel to the skin. A: SWE examination of the lesion is shown. Five different SWV measurements were obtained from the central part of the lesion and 1 SWV measurement was obtained from the adjacent fat tissue. B: SE of the lesion is shown. Calculation of the SR was based on the comparison of the average strain of the breast mass and the fat tissue. The ROI, expressed as T, was placed on the lesion to include a greater amount of it, and the ROI, expressed as R, was placed on the adjacent normal fat tissue. The SR was calculated as the ratio of R to T (R/T). The lesion underwent a core needle biopsy and was diagnosed as a fibroadenoma

SWE examinations were performed using the Virtual Touch Tissue Imaging Quantification (VTIQ) function. The probe was gently placed perpendicular to the skin with no applied pressure, and enough gel was used to avoid a compression effect. Imaging was performed in the longitudinal plane of the lesion. After the VTIQ function was triggered, the lesion was included in a rectangular region of interest (ROI) elasticity box. The ROI box was placed to ensure that both the whole lesion and sufficient surrounding fat tissue were included. A 2-dimensional (2D) elastography color map was displayed on the screen. For each lesion, 3–5 small ROI boxes were randomly placed depending on the lesion’s size (Figure 1a). Lesion stiffness was calculated as the shear wave velocity (SWV) in m/s. One SWV measurement was also obtained from the adjacent fat tissue. Shear wave quality maps were obtained for each examination on which high-quality regions were displayed as green and low-quality regions were displayed as orange. All SWV measurements were obtained from the green areas on the shear wave quality map. The maximum SWV, maximum SWV to fat tissue SWV ratio, the mean SWV, and the mean SWV to fat tissue SWV ratio were used for statistical analysis.

### 2.4. Strain elastography

SE examinations were performed using an 9-MHz probe on an Aplio 500 US machine (Toshiba Medical Systems, Otawara, Japan). The US probe was again gently held perpendicular to the skin and a sonoelastographic ROI box was placed on the lesion, including sufficient fat tissue. Five or six compressive and decompressive forces were applied in an antero–posterior direction. Compressive and decompressive waves were seen above and below the baseline on the elastography screen. Strain measurements were performed when the appropriate sinusoidal shape relaxation wave was obtained. Calculation of the SR was based on the comparison of the average strain of the breast mass and the fat tissue. The ROI, expressed as T, was placed on the lesion to include a large amount of the lesion, and the ROI, expressed as R, was placed on the adjacent normal fat tissue at the same level with the lesion. The SR was calculated as the ratio of R to T (R/T) (Figure 1b).

### 2.5. Biopsy procedure and histopathological examinations

All breast lesions included in this study underwent US-guided core needle biopsies with a 14 G biopsy needle (22-mm excursion; Geotek, Maxicore, Ankara, Turkey). The final diagnosis was based on histopathological results.

### 2.6. Statistical analysis

Statistical analysis was performed with the Statistical Package for Social Sciences 22.0 (SPSS Inc., Chicago, IL, USA) for Windows. A P-value less than 0.05 was accepted as statistically significant. Categorical variables were expressed in frequencies and compared with chi-square tests. Continuous variables are presented as mean ± standard deviation (SD), median, and range, as appropriate. Kolmogorov–Smirnov tests were used to assess normal distributions of the quantitative data. Mann–Whitney U tests and Student’s t-tests were used to compare elasticity values of the benign and malignant lesions, as appropriate.

Receiver operating characteristic (ROC) curves were constructed to assess the diagnostic performance of the B-mode US, SWE, and SE. The optimal cutoff values were obtained by maximizing the Youden index (Youden index = sensitivity + specificity – 1). The sensitivity, specificity, positive predictive value (PPV), negative predictive value (NPV), and accuracy for each diagnostic technique were calculated, and sensitivity and specificity values were compared using McNemar tests. Lesions were categorized according to the optimal cutoff values for each elastographic technique as a test positive or test negative. As the highest AUC value for SWE was obtained from the maximum SWV measurement, we used the maximum SWV and SR to compare consistent and discrepant findings in both techniques.

## 3. Results

### 3.1. Demographic and pathological results

**Figure 2:**
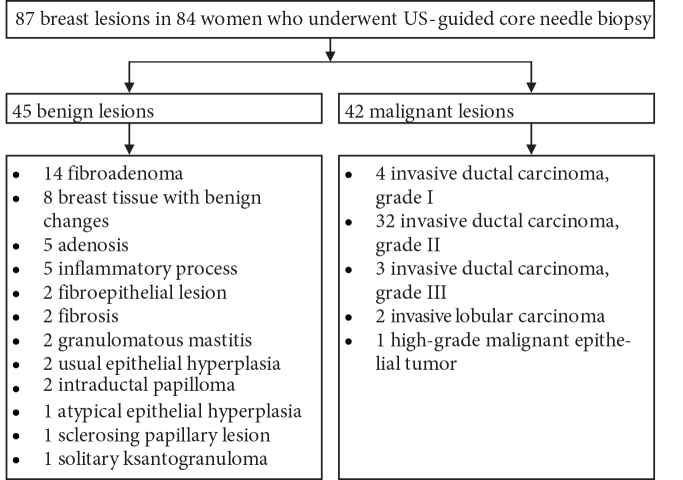
Lesions according to the pathology results are shown.

A total of 87 breast masses were examined in 84 consecutive women. Eighty-one women had a single breast mass, while 3 women had double breast masses. Of the 87 lesions, 49 (56.3%) were in the left breast (27 malignant vs. 22 benign) and 38 (43.7%) were in the right breast (15 malignant vs. 23 benign). The maximum lesion diameter ranged from 5 to 73 mm (mean ± SD: 20.22 ± 12.68 mm). Pathology results revealed 42 malignant (48.3%) and 45 benign (51.7%) histologies (Figure 2).

### 3.2. B-mode US

B-mode US examinations revealed 16 (18.4%) BIRADS category 3, 33 (37.9%) BIRADS category 4, and 38 (43.7%) BIRADS category 5 lesions. All BIRADS category 3 lesions were diagnosed with a benign pathology. These lesions underwent biopsy because of the surgeon’s or patient’s request, or because the patients were at high risk. Thirty-five of the BIRADS category 5 lesions and 7 of the BIRADS category 4 lesions were diagnosed with a malignant pathology (Table 1). The area under the curve (AUC) for the ROC analysis for the BIRADS category was 0.796. The dichotomized sensitivity, specificity, accuracy, PPV, and NPV values were 100% (42/42), 35.6% (16/45), 66.7% (58/87), 59% (42/71), and 100% (16/16), respectively. The mean maximum size of the malignant lesions was 21.93 ± 14.05 mm (range: 7–73 mm) and the mean maximum size of the benign lesions was 18.62 ± 11.17 mm (range 5–50 mm) (P = 0.127).

**Table 1:** BIRADS categories of the lesions are shown.

BIRADS Category	BIRADS 3	BIRADS 4	BIRADS 5
Benign pathology	16	26	3
Malignant pathology	0	7	35
Sensitivity	100%	21%	92%
Specificity	NA	NA	NA
P-value	< 0.001	<0.001	< 0.001

BIRADS: Breast Imaging Reporting and Data System.NA: Not applicable.

### 3.3. Comparison of diagnostic performances of shear wave elastography and strain elastography

**Figure 3:**
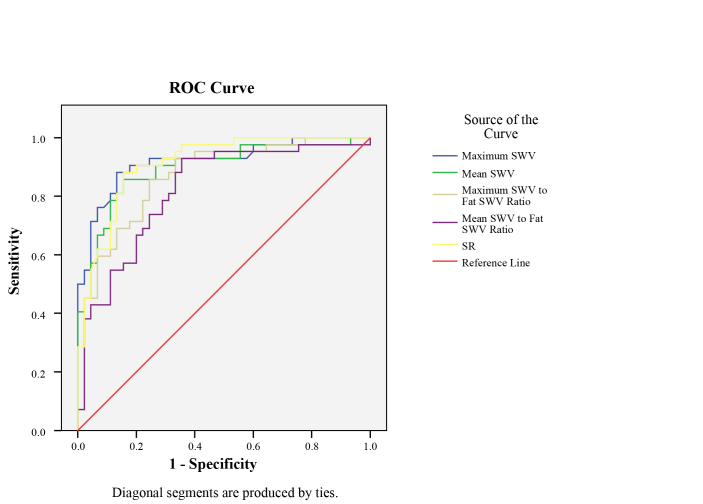
The ROC curve analysis of the measurements in the differentiation of malignant and benign lesions is shown. The maximum SWV and SR had the highest diagnostic performance (AUC: 0.918 [95% confidence interval (CI): 0.858–0.978], P< 0.001; AUC: 0.913 [95% confidence interval (CI): 0.854–0.971], P< 0.001, respectively).

On SWE, the maximum SWV, maximum SWV to fat tissue SWV ratio, the mean SWV, the mean SWV to the fat tissue SWV ratio, and the SR of the malignant lesions significantly differed from those of the benign lesions (Table 2). ROC curves for assessing the diagnostic performance of each elastography method is shown in Figure 3. The AUC, sensitivity, specificity, accuracy, PPV, and NPV for the best cutoff values are displayed in Table 3. The highest AUC values belonged to the maximum SWV and SR (AUC: 0.918 and AUC: 0.913, respectively). The diagnostic performance of the maximum SWV, mean SWV, and maximum SWV to fat SWV ratio were similar to the diagnostic performance of the SR (P = 1.00, P = 1.00, P = 0.629, respectively). However, the diagnostic performance of the mean SWV to fat SWV ratio was significantly lower than that of the SR (P = 0.013).

**Table 2:** Elastography values of the benign and malignant lesions are shown.

	Elasticity values		
Elastography method	Benign	Malignant	P-value
Maximum SWV (m/s)	2.58 ± 0.83 (1.22–4.87)	4.90 ± 1.48 (2–9)	<0.001
Mean SWV (m/s)	2.33 ± 0.75 (1.11–4.26)	4.13 ± 1.39 (1.34–8.43)	<0.001
Max SWV to fat SWV ratio	1.68 ± 0.54 (1.07–3.98)	2.65 ± 0.81 (1.27–4.64)	<0.001
Mean SWV to fat SWV ratio	1.52 ± 0.50 (0.94–3.70)	2.24 ± 0.76 (0.86–4.13)	<0.001
SR	2.19 ± 1.47 (0.50–7.50)	7.12 ± 5.78 (1.68–33.20)	<0.001

Data are mean ± SD (range). SWV: Shear wave velocity; SR: Strain ratio.

**Table 3:** The AUC, sensitivity, specificity, accuracy, PPV, and NPV for the best cutoff values of each examination are shown.

Elastography Method	AUC (CI)	Cutoff	Sensitivity (%)	Specificity (%)	Accuracy (%)	PPV (%)	NPV (%)	P-valuea
Max SWV	0.918(0.858–0.978)	3.41	37/42 (88.1)	39/45 (86.7)	76/87 (87.4)	37/43 (86.1)	39/44 (88.6)	1.00
Mean SWV	0.895(0.826–0.964)	2.98	36/42 (85.7)	38/45 (84.4)	74/87 (85.1)	36/43 (83.7)	38/44 (86.4)	1.00
Max SWV to fat SWV ratio	0.866(0.789–0.942)	1.91	36/42 (85.7)	34/45 (75.6)	70/87 (80.5)	36/47 (76.6)	34/40 (85.0)	0.629
Mean SWV to fat SWV ratio	0.823(0.734–0.912)	1.51	39/42 (92.9)	29/45 (64.4)	68/87 (78.2)	39/55 (70.9)	29/32 (90.6)	0.013
SR	0.913(0.854–0.971)	3.22	37/42 (88.1)	38/45 (88.4)	75/87 (86.2)	37/44 (84.1)	38/43 (88.4)	-

a: P-values derived from comparison of the row value with the SR; AUC: Area under curve; CI: Confidence interval; SR: Strain ratio; SWV: Shear wave velocity; NPV: Negative predictive value; PPV: Positive predictive value.

The best cutoff maximum SWV to differentiate benign lesions from malignant ones was 3.41 m/s. According to this cutoff value, 6/45 lesions (1 fibrosis, 2 intraductal papillomas, 1 adenosis, 1 solitary xanthogranuloma, and 1 fibroadenoma) were false-positive and 5/42 lesions (1 high-grade malignant epithelial tumor and 4 invasive ductal carcinomas) were false-negative. Five of the false-positive lesions were categorized as BIRADS category 4, and 1 false-positive lesion was categorized as BIRADS category 3. All false-negative lesions belonged to BIRADS category 5. The maximum lesion size did not differ significantly in false-negative and false-positive groups (19.27 ± 12.41 mm vs. 20.36 ± 12.79 mm, P = 0.725).

The best cutoff value of SR for the differentiation of benign lesions from malignant ones was 3.22. According to this value, 7/45 lesions (1 solitary xanthogranuloma, 1 inflammatory process, 3 fibroadenomas, 1 intraductal papilloma, and 1 fibroepithelial lesion) were false-positive and 5/42 lesions (1 high-grade malign epithelial tumor and 4 invasive ductal carcinomas) were false-negative. Six false positive lesions were categorized as BIRADS category 4, and 1 false-positive lesion was categorized as BIRADS category 3. Four of the false-negative lesions belonged to BIRADS category 5 and 1 false-negative lesion belonged to BIRADS category 4. The maximum lesion size did not differ significantly in the false-negative and false-positive groups (26.42 ± 18.10 mm vs. 19.23 ± 11.44 mm, respectively, P = 0.106).

### 3.4. Comparison of discrepant and consistent findings in shear wave and strain elastography

In 17 lesions, SWE and SE showed discrepant results (Table 4). One of these lesions was a BIRADS category 3, 9 were BIRADS category 4, and 7 were BIRADS category 5. For the benign lesions with discrepant results, SWE diagnosed 5 lesions accurately and SE diagnosed 4 lesions accurately. For the malignant lesions with discrepant results, SWE diagnosed 4 lesions accurately and SE diagnosed 4 lesions accurately.

**Table 4:** Comparison of discrepant findings on SWE and SE is shown.

Case number	Pathology	Correct diagnosis	BIRADS category	Max SWV	SR	Max lesion size (mm)
1	FA	SWE	4	2.80	3.24	38
2	FA	SWE	4	3.06	7.50	46
3	FA	SE	4	3.92	3.04	12
4	FA	SWE	4	3.05	5.81	21
5	Intraductal papilloma	SE	4	4.14	3.07	8
6	Fibroepithelial lesion	SWE	4	2.89	3.94	33
7	Adenosis	SE	3	3.66	2.07	39
8	Fibrosis	SE	4	4.87	3.20	35
9	Mastitis	SWE	4	1.69	4.67	13
10	IDC	SWE	5	4.36	2.55	19
11	IDC	SWE	5	5.29	1.68	70
12	IDC	SE	5	2.33	4.26	46
13	IDC	SE	5	3.09	4.67	20
14	IDC	SE	5	2.34	5.14	9
15	IDC	SE	5	3.00	3.23	16
16	IDC	SWE	4	3.94	2.30	11
17	IDC	SWE	5	4.00	3.11	19

BIRADS: Breast Imaging Reporting and Data System; FA: Fibroadenoma; IDC: Invasive ductal carcinoma; SR: Strain ratio; SWE: Shear wave elastography; SWV: Shear wave velocity.

In 67 lesions, both SWE and SE showed correct results with the pathology examination. One malignant lesion (a high-grade malignant epithelial tumor) was diagnosed as false-negative on both SWE and SE, and 2 benign lesions (1 intraductal papilloma and 1 solitary xanthogranuloma) were diagnosed false positive on both SWE and SE.

## 4. Discussion

Our study results confirmed that both SWE and SE are capable of differentiating benign and malignant breast lesions. The two techniques had similar diagnostic performances. The maximum SWV and SR of the lesions had the highest diagnostic performance (AUC = 0.918, AUC = 0.913, respectively).

Malignant breast lesions tend to be stiffer than benign ones; this paradigm constitutes the basis of an elastography examination. Several studies have demonstrated that both SWE and SE have the ability to differentiate benign breast masses from malignant ones [2,3,18–24]. In a study evaluating the diagnostic performance of SE by Thomas et al., the sensitivity and specificity were 90% and 89%, respectively. In addition, when compared with B-mode, the specificity of SE was higher at a SR cutoff value of 2.45 (56% vs. 89%) [20]. In a study by Zhi et al. [21], a cutoff value of 3.05 for SR yielded a 92.4% sensitivity and a 91.1% specificity. Zhao et al. [22] reported that the cutoff SR value of 3.06 for the differentiation of malignant and benign lesions led to an 87.7% sensitivity and an 88.5% specificity. Balcik et al. [3] reported the sensitivity and specificity of SE as 85.5% and 84.8% at a SR threshold value of 4.55. In our study, the optimal cutoff SR value was 3.22, and this yielded 88.1% sensitivity and 84.4% specificity, which was concordant with previous studies.

In the literature, studies have reported using the VTIQ method of SWE for the evaluation of breast lesions [2,23–29]. Ianculescu et al. [2] reported the sensitivity and specificity values of the VTIQ method as 80.4% and 73%, respectively, when the cutoff SWV was 3.31 m/s. In addition, when VTIQ was combined with B-mode US, diagnostic sensitivity and specificity were increased (92% and 72.9%, respectively). Tang et al. [23] found the sensitivity and specificity of the VTIQ technique as 93.3% and 79.4%, respectively, at a mean SWV cutoff value of 3.68 m/s. Golatta et al. [24] reported that a cutoff value of 5.18 m/s led to sensitivity and specificity values of 98% and 68%, respectively. In other studies, cutoff SWV values for SWE examination ranged from 6.593 m/s to 3.23 m/s [24–29]. Magalhaes et al. [26] measured SWV of the lesions from the stiffest part seen on the elastography color map, which may explain the high cutoff value of their study. In our study, the maximum SWV of the malignant lesions was 4.90 ± 1.48 m/s and the maximum SWV of the benign lesions was 2.58 ± 0.83 m/s. The optimal maximum SWV cutoff value was 3.41 m/s, which was concordant with the previous studies.

There are a limited number of studies that have compared the diagnostic performance of SWE with SE (Table 5). In the study by Chang et al. [13], the sensitivity of SWE was higher than that of SE, and the specificity of SE was higher than that of SWE. However, overall diagnostic performances of those two elastography techniques were similar. In this study, the diagnostic performance of SE with a 5-point scoring system compared with shear wave measurements. The difference in our study from the Chang et al. study was that we performed 4 different calculations using SWV values and we used SR instead of 5-point scoring system. Among these calculations, the maximum SWV had the highest AUC value. A comparison of the maximum SWV with SE demonstrated no significant difference in the differentiation of benign and malignant breast lesions. Barr and Zhang [14] reported higher diagnostic performance of SE than SWE. In their study, for the SWE examination, 3 measurements were performed from the lesion, and the maximum values were used for statistical analysis. In the SE technique, the ratio of the longest diameter of the lesion on elastography to the longest diameter of the lesion on B-mode sonography (E/B) was used for statistical analysis. However, their study mainly focused on if the quality measure (QM) of SWE increased the diagnostic performance compared to SWE without QM, and they did not compare the AUC values of SWE and SE techniques. For SWE with QM, the optimal cutoff value of 4.5 m/s led to a 93% sensitivity and an 89% specificity. For SE, the optimal E/B cutoff value of 1 led to a 98% sensitivity and an 87% specificity. In our study, the sensitivity and specificity of both techniques were lower when compared to Barr et al.’s study. However, these two studies used relatively different elastography techniques. Seo et al. [12] reported similar diagnostic performances for both SWE and SE. In their study, the sensitivity of SR (95%) was higher than the mean elasticity (85%), and the specificity of the mean elasticity (96%) was higher than the SR (84%); however, the difference was not statistically significant.

**Table 5:** Previously published studies comparing shear wave and strain elastography are shown.

	AUC		Sensitivity (%)		Specificity (%)		P-value a	Comments
Study	SWE	SE	SWE	SE	SWE	SE		
Chang et al.2013	0.928	0.943	95.8	81.7	84.8	93.7	0.503	Authors assessed the diagnostic performance of SE with a 5-point scoring system and compared with shear wave measurements. Overall diagnostic performances of those two elastography techniques were similar.
Barr et al.2014	…	0.990	93	98	89	87	…	Authors assessed diagnostic performance of SE, SWE without QM, and SWE with QM. Addition of QM to SWE improved sensitivity significantly. However, authors did not compare AUC of SE and SWE with QM.
Seo et al. 2018	0.898	0.929	85	95	96	84	0.490	Authors compared SR and mean elasticity (maximum stiffness of target/stiffness of fat). No significant difference was found between the two elastography techniques.
Youk et al. 2014	0.907	0.917	71.4	76.2	100	81.0	0.077	SWE measurements were calculated as mean elasticity, maximum elasticity, and elasticity ratio. Maximum AUC belonged to elasticity ratio. All AUC values of SWE were not statistically different than AUC of SR.

a Derived from the comparison of AUC of SWE and SE. AUC: Area under curve; SWE: Shear wave elastography; SE: Strain elastography; QM: Quality measure; SR: Strain ratio.

Youk et al. [15] compared the diagnostic performance of SWE and SE. For SWE, they calculated maximum elasticity, mean elasticity, and elasticity ratio and compared these variables with SR. Their results demonstrated no statistically significant difference between any SWE calculations and SR. The difference in our study was that comparison of mean SWV to fat SWV ratio showed a significantly lower diagnostic performance than SR. Kim et al. [1] applied SWE and SE on the same breast lesions and combined B-mode US findings with the SWE and SE findings. The combination of B-mode US, SWE, and SE yielded higher specificity, accuracy, and PPV than B-mode US alone. In their study, both SWE and SE succeeded in differentiating benign and malignant lesions; however, the authors did not compare the diagnostic performance of each elastography technique.

Although the diagnostic performance of SWE and SE were similar in our study, we had 17 cases with discrepant results. Of these cases, SWE had a correct diagnosis in 9 cases, and SE had a correct diagnosis in 8 cases. With the SWE technique, 6/45 benign lesions had a false-positive diagnosis and 5/42 malignant lesions had a false-negative diagnosis. With the SE technique, 7/45 benign lesions had a false-positive diagnosis and 5/42 malignant lesions had a false-negative diagnosis. In 2 cases (1 solitary xanthogranuloma and 1 intraductal papilloma), both SWE and SE revealed a false-positive diagnosis. There was only 1 case (1 high-grade malignant epithelial tumor) that both SWE and SE revealed a false-negative diagnosis.

Our study had some limitations. First, we studied a limited population in number. Second, we did not assess the interobserver variability. Third, we did not assess the 5-point color scale of the strain elastography examinations. However, we focused on the quantitative or semiquantitative measurements of SWE and SE. Fourth, we used different vendor machines for SWE and SE, as each vendor machine in our study was not able to perform the other elastography technique. 

In conclusion, our study confirmed that both SE and SWE are feasible imaging modalities in the differentiation of malignant and benign breast lesions with similar diagnostic performances.

## References

[ref1] (2014). Diagnostic value of elastography using acoustic radiation force impulse imaging and strain ratio for breast tumors. Journal of Breast Cancer.

[ref7] (2014). Added value of Virtual Touch IQ shear wave elastography in the ultrasound assessment of breast lesions. European Journal of Radiology.

[ref12] (2016). Efficacy of sonoelastography in distinguishing benign from malignant breast masses. The Journal of Breast Health.

[ref20] (2017). The diagnostic value of shear wave elastography for parathyroid lesions and comparison with cervical lymph nodes. Medical Ultrasonography.

[ref26] (2017). Evaluation of renal parenchyma elasticity with acoustic radiation force impulse quantification in nutcracker syndrome and comparisons with grayscale doppler sonography and laboratory findings. Journal of Ultrasound in Medicine.

[ref33] (2014). Comparison of muscle-to-nodule and parenchyma-to-nodule strain ratios in the differentiation of benign and malignant thyroid nodules: Which one should we use?. European Journal of Radiology.

[ref39] (2007). Imaging and estimation of tissue elasticity by ultrasound. Ultrasound Quarterly.

[ref45] (2012). Combination of elastography and tissue quantification using the acoustic radiation force impulse (ARFI) technology for differential diagnosis of breast masses. Japanese Journal of Radiology.

[ref49] (2007). Differentiating benign from malignant solid breast masses with US strain imaging. Radiology.

[ref55] (2011). Breast mass evaluation: factors influencing the quality of US elastography. Radiology.

[ref61] (2017). Value of shear wave elastography in discriminating malignant and benign breast lesions: A meta analysis. Medicine.

[ref67] (2018). Comparison and combination of strain and shear wave elastography of breast masses for differentiation of benign and malignant lesions by quantitative assessment: preliminary study. Journal of Ultrasound in Medicine.

[ref73] (2013). Comparison of shear-wave and strain ultrasound elastography in the differentiation of benign and malignant breast lesions. AJR American Journal of Roentgenology.

[ref77] (2014). Shear-wave elastography of the breast: value of a quality measure and comparison with strain elastography. Radiology.

[ref83] (2014). Comparison of strain and shear wave elastography for the differentiation of benign from malignant breast lesions, combined with B-mode ultrasonography: qualitative and quantitative assessments. Ultrasound in Medicine &amp; Biology.

[ref89] (2015). STARD 2015: an updated list of essential items for reporting diagnostic accuracy studies.. Radiology.

[ref96] (2013). Breast Imaging Reporting and Data System.

[ref105] (2007). Differentiating benign from malignant solid breast masses with US strain imaging. Radiology.

[ref111] (2012). Shear wave elastography for breast masses is highly reproducible. European Radiology.

[ref117] (2010). Significant differentiation of focal breast lesions: calculation of strain ratio in breast sonoelastography. Academic Radiology.

[ref121] (2010). Ultrasonic elastography in breast cancer diagnosis: strain ratio vs 5-point scale. Academic Radiology.

[ref126] (2012). Diagnosis of solid breast lesions by elastography 5-point score and strain ratio method. European Journal of Radiology.

[ref130] (2015). A novel two-dimensional quantitative shear wave elastography for differentiating malignant from benign breast lesions. International Journal of Clinical and Experimental Medicine.

[ref137] (2014). Evaluation of virtual touch tissue imaging quantification, a new shear wave velocity imaging method, for breast lesion assessment by ultrasound. BioMed Research International.

[ref143] (2013). Shear wave velocity measurements for differential diagnosis of solid breast masses: a comparison between virtual touch quantification and virtual touch IQ. Ultrasound in Medicine &amp; Biology.

[ref149] (2017). Diagnostic value of ARFI (Acoustic Radiation Force Impulse) in differentiating benign from malignant breast lesions.. Academic Radiology.

[ref157] (2017). Clinical application of acoustic radiation force impulse imaging with virtual touch IQ in breast ultrasound: diagnostic performance and reproducibility of a new technique.. Acta Radiologica.

[ref163] (2016). Value of virtual touch tissue imaging quantification for evaluation of ultrasound breast imaging-reporting and data system Category 4 lesions.. Ultrasound in Medicine &amp; Biology.

[ref169] (2016). Combination of two-dimensional shear wave elastography with ultrasound breast imaging reporting and data system in the diagnosis of breast lesions: a new method to increase the diagnostic performance. European Radiology.

